# Shortening time for access to alcohol drives up front-loading behavior, bringing consumption in male rats to the level of females

**DOI:** 10.1186/s13293-021-00395-y

**Published:** 2021-09-15

**Authors:** Annabelle Flores-Bonilla, Barbara De Oliveira, Andrea Silva-Gotay, Kyle W. Lucier, Heather N. Richardson

**Affiliations:** 1 Neuroscience and Behavior Program, The University of Massachusetts Amherst, Amherst, MA 01003 USA; 2 Department of Psychological and Brain Sciences, The University of Massachusetts Amherst, Amherst, MA 01003 USA

**Keywords:** Sex differences, Binge drinking, Alcohol, Operant self-administration, Front-loading

## Abstract

**Background:**

Incentives to promote drinking (“happy hour”) can encourage faster rates of alcohol consumption, especially in women. Sex differences in drinking dynamics may underlie differential health vulnerabilities relating to alcohol in women versus men. Herein, we used operant procedures to model the happy hour effect and gain insight into the alcohol drinking dynamics of male and female rats.

**Methods:**

Adult male and female Wistar rats underwent operant training to promote voluntary drinking of 10% (w/v) alcohol (8 rats/sex). We tested how drinking patterns changed after manipulating the effort required for alcohol (fixed ratio, FR), as well as the length of time in which rats had access to alcohol (self-administration session length). Rats were tested twice within the 12 h of the dark cycle, first at 2 h (early phase of the dark cycle, “early sessions”) and then again at 10 h into the dark cycle (late phase of the dark cycle, “late sessions”) with an 8-h break between the two sessions in the home cage.

**Results:**

Adult females consumed significantly more alcohol (g/kg) than males in the 30-min sessions with the FR1 schedule of reinforcement when tested late in the dark cycle. Front-loading of alcohol was the primary factor driving higher consumption in females. Changing the schedule of reinforcement from FR1 to FR3 reduced total consumption. Notably, this manipulation had minimal effect on front-loading behavior in females, whereas front-loading behavior was significantly reduced in males when more effort was required to access alcohol. Compressing drinking access to 15 min to model a happy hour drove up front-loading behavior, generating alcohol drinking patterns in males that were similar to patterns in females (faster drinking and higher intake).

**Conclusions:**

This strategy could be useful for exploring sex differences in the neural mechanisms underlying alcohol drinking and related health vulnerabilities. Our findings also highlight the importance of the time of testing for detecting sex differences in drinking behavior.

## Highlights

- Voluntary alcohol drinking is higher in adult female rats compared to adult male rats. This sex difference is most pronounced in the later phase of the dark cycle, and when the operant effort is minimal (when 1 lever press gives 1 reward: fixed ratio 1, FR1).
- Higher alcohol intake in females is primarily due to “front-loading”, or the rapid consumption of alcohol within the first 5 min of access.
- Increasing the effort required to obtain alcohol from FR1 to FR3 dampens front-loading drinking behavior, resulting in similar levels of total intake in males and females.
- Compressing the time of access to 15 min drives up front-loading to such a degree that rats end up consuming more alcohol in total than they do in 30-min sessions. In males, this increase in drinking is large enough that it eliminates the sex difference in total alcohol intake.

## Background

Alcohol use disorder (AUD) is a chronic relapsing condition characterized by the misuse of alcohol [1–3]. In the United States (US), 29% of adults have a lifetime prevalence for AUD, and men have a higher prevalence than women, yet this gap has been closing in recent years [3–5]. Serious long-term effects of AUD are presented differently in men and women even when drinking levels are comparable, with higher risks of alcohol-related liver disease, cancer, cardiovascular disease, and brain damage in women compared to men [5–12]; reviewed in [13–17]. The damaging effects of alcohol on the liver and brain are exacerbated by excessive patterns of compulsive drinking, e.g., binge drinking [18–23]; reviewed in [24–29]. Binge drinking is considered the first of the three stages of addiction: binge/intoxication, negative affect/withdrawal, and preoccupation/anticipation, with evidence of sex differences being reported in all three stages, reviewed in [26, 29]. While the prevalence of binge drinking in the US is higher in men (33%) than women (17%), women experience greater mental health instability (17.3% versus 8.6%) and worse mental health consequences (11.2% versus 6.3%) compared to men [30, 31]. Incentives can alter the drinking dynamics of alcohol consumption in humans, such as “happy-hour” [32, 33]. Women are 1.45 times more likely to report experiencing altered drinking (drinking more, drinking more quickly, or both) during happy hour events than men, which may be due to receiving drinks rather than buying them and/or as a consequence of being targeted for gender-specific specials such as “ladies’ night” or “ladies’ happy hour” [34].

As a model of voluntary alcohol consumption seen in humans, rodents in the laboratory are useful for maintaining the experimental control needed to understand the behaviors leading to changes in alcohol consumption and study its effects in the brain. A previous report using an operant model in male rats showed that reducing session length in which rats could press a lever to receive alcohol had increased the total amount of alcohol consumed [35]. However, it is currently unknown whether higher intake was due to changes in the drinking dynamics within the session. For example, others have described “front-loading” as a burst of rapid drinking behavior at the start of the session when rodents first have access to alcohol [35–48]. Front-loading is found to be specific to rewarding substances, as mice that previously exhibited front-loading behavior to alcohol did not show this same behavioral response for water [45–47]. Other rewarding substances are capable of inducing front-loading in mice and rats, including sweet high-fat liquids and sodium solution to sodium-depleted rats, which makes front-loading behavior a consistent behavioral phenotype for seeking and consuming rewards [37, 38]. In addition, front-loading has implications for the study of alcohol drinking behavior in rodents as a model for human binge drinking.

Modeling a happy hour in rodents is a powerful tool that can be used to determine how incentivizing voluntary alcohol drinking impacts specific patterns of drinking behaviors (drinking more, drinking more quickly, or both) in males versus females. Neither front-loading drinking behavior nor the happy hour effect on this has been well-characterized in female rats. Operant self-administration in rodents allows the precise tracking and manipulation that can address these questions [49–56]; reviewed in [57, 58]. We used this approach to map out the dynamic microstructural patterns in drinking in adult male and female rats during both a standard and a happy hour session. Female rodents drink more alcohol than males in adulthood, and these experiments were designed specifically to test the hypothesis that differences in drinking dynamics between males and females lead to different levels of alcohol consumption [42, 59–63]. Additionally, we also investigated how these patterns of drinking are expressed when rats are given a reduced amount of time to drink (happy hour).

## Materials and methods

### Animals

A total of 24 adult Wistar rats (12 per sex) were obtained from Charles River (Wilmington, MA, USA). Upon arrival, the rats were 85 days old and group-housed by sex (2 rats per cage) under a reverse light cycle of 12-h light/12-h dark (lights OFF at 7 am/lights ON at 7 pm). Cages were standard plastic with wood chip bedding and rats had food and water access ad libitum with no deprivation. Rats acclimated for 15 days before operant training began. Rats were weighed once a day and weight data was used to calculate the alcohol intake per weight (g/kg) of each rat. At the start of the experiment, males weighed 352.00 ± 5.42 g and females weighed 241.25 ± 4.81 g. All procedures were performed following the “Guide for the Care and Use of Laboratory Animals” prepared by the Institute of Laboratory Animal Resources, National Academy of Sciences and approved by the Institutional Animal Care and Use Committee of the University of Massachusetts Amherst.

### Voluntary operant self-administration experiments

#### Apparatus: operant conditioning chambers

Operant training and subsequent testing were conducted in computer-controlled operant conditioning chambers [64, 65]. One of the levers was active, in which a response (active lever press) consequently delivered 0.1 ml of fluid from a 15 rpm Razel syringe pump (Stamford, CT) to the appropriate well over 0.5 s. The fluid delivered was tap water in the 12-h training session and either water (control) or 10% (w/v) alcohol (ethanol) diluted in tap water (alcohol group) in subsequent training and testing sessions. The alternate lever was inactive, only recording the operant response (inactive lever press) without delivery of fluid, used as a measure of “non-discriminative lever pressing” [66]. The active lever was programmed to a fixed ratio 1 (FR1) schedule for training and its location was counterbalanced between each rat (half of the rats had an active left lever, and the other half had an active right lever). All lever presses and subsequent deliveries were recorded using custom Med-PC IV software. Both levers were available from the start of the session and did not retract until the end of the session. After each operant session, the drinking apparatus was inspected for any leftover liquid to confirm rats had consumed all the alcohol (or water for control rats). This occurred only once in which there was alcohol left in the cup and it overflowed into the bedding. This session for this rat was excluded from the data analysis. Rats also had ad libitum access to food and water during all the operant sessions (a ceramic or stainless-steel dish containing rat chow and a water bottle were always available in each operant box). This ensured that lever presses for alcohol were not motivated by hunger or thirst.

#### Operant training

**Fig. 1:**
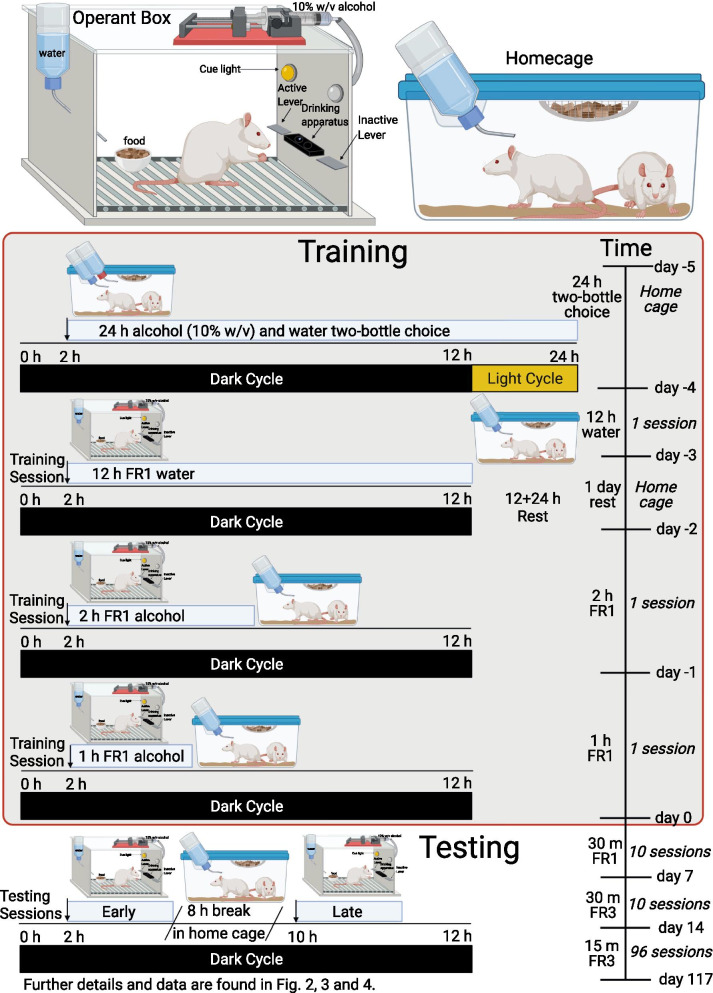
Operant conditioning boxes and experimental timeline. Male and female adult Wistar rats were trained to self-administer 10% w/v alcohol using operant conditioning. Training began with 1 day of two-bottle choice in their home cages, followed by the first day of operant training for water (12 h session), a day of rest in the home cage, and two days of operant training for alcohol (2 h session on the first day and 1 h session on the second day). Operant testing was conducted twice each day, beginning at 2 h (early session) and 10 h (late session) in the dark cycle for a total of 117 days. Operant testing was conducted in normal sessions of minimal effort (30 m fixed ratio 1), normal sessions of increased effort (30 m fixed ratio 3) and compressed happy hour sessions of increased effort (15 m fixed ratio 3) which are summarized in the timeline. Operant boxes had two levers, one active and one inactive, as well as a food cup and water bottle that could be accessed ad libitum. Alcohol was delivered to a two-well acrylic drinking apparatus after every 1 press (FR1) or 3 presses (FR3). The cue light above the active lever is turned on for each alcohol delivery. *w/v* weight per volume, *FR* fixed ratio, *h* hour, *m* minutes. Created with BioRender.com

Rats were randomly assigned to an alcohol group (16 rats; 8 per sex) or water control group (8 rats; 4 per sex) before the start of the training. A detailed timeline of experiments can be found in Fig. 1. To reduce novelty of the alcohol solution, on the first day of training rats had two-bottle choice (2BC) access to 10% (w/v) alcohol (ethanol) diluted in tap water and only tap water in their home cage for 24 h [67]. To initiate operant training, the next day rats were trained for 12 h to orally self-administer water in operant boxes. After that, rats were placed in their home cage for a full additional 24 h to rest. On the fourth day of training, all rats were placed in the operant boxes for 2 h of access to 10% (w/v) alcohol or tap water. The next day, time in the operant box was reduced to 1 h. The operant training sessions were conducted once a day and started after the first 2 h of the dark cycle (at 9 am). To progress from operant training to testing, rats had to lever press at least once in one of the training sessions.

### Manipulation of operant parameters

At the start of the drinking testing sessions the following day, rats were placed in the operant boxes twice a day: once at the start of the dark cycle (9:00 am) and a second time at the end of the dark cycle (5:00 pm) with an 8-h break in the home cage between the sessions (~ 9:30 am–5:00 pm in home cage). This is adapted from a previously described alcohol operant self-administration method where male rats had access to the operant chambers twice in the light cycle (once in the morning and once at the end of the afternoon) in which they found no differences in the number of lever presses performed for alcohol [35].

*Fixed ratio 1 schedule (30-min sessions)*: We first characterize alcohol drinking patterns in male and female rats. Testing sessions were conducted in the operant chambers with several changes in operant parameters of alcohol accessibility and data was collected as lever presses per minute of the session (in 1-min bins) and as total lever presses per session. In the first five days, rats had 30-min access in an FR1 schedule (“normal effort”) for a total of 10 sessions and two no-drinking (rest) days in their home cage.

*Fixed ratio 3 schedule (30-min sessions):* We next studied whether an increase in effort to obtain alcohol affected alcohol drinking patterns in males and females. We changed the number of lever presses required for an alcohol delivery from 1 to 3 (FR3 schedule) for the next 10 sessions, followed by two rest days in their home cage.

*Fixed ratio 3 schedule (15-min sessions):* Finally, to test whether limiting access time could increase drinking speed, and if sensitivity to this manipulation differed with sex, we reduced the length of operant sessions from 30 min (“normal access”) to 15 min (“compressed access”). This manipulation has previously been used in male rats [35] to model alcohol-incentivizing events such as happy hour in humans [32–34]. Rats continued with daily testing for 6 weeks (2 sessions per day for 3–5 consecutive days each week). We then switched to testing rats every other day (2 sessions per day) to determine if voluntary alcohol intake could be augmented by an intermittent testing schedule, similar to earlier reports [68, 69].

### Statistical analysis

Statistical analyses and the generation of graphs were done using GraphPad Prism version 8.4.3 for Mac (GraphPad Software, San Diego, CA). Data are shown as the mean and standard error of the mean (± SEM) unless otherwise indicated. Total alcohol intake (g/kg), total water intake (ml/kg), responding on the active lever (presses/min) and responding on the inactive lever (presses/min) data in each within-subject condition (minimal effort versus increased effort and normal access versus compressed access) were analyzed using a two-way mixed model analysis of variance (MM-ANOVA) with session (early session versus late session) as the within-subject factor and sex (male versus female) as the between-subject factor. The intake was confirmed by evidence of no leftover liquid after each session. Bonferroni’s multiple comparisons test was conducted as post hoc analysis following significant main effects or interactions, as recommended [70, 71].

For the analysis of sex differences in alcohol microstructural drinking behavior, the alcohol intake data (g/kg) in 1-min bins were analyzed using a three-way, sex × session × time MM-ANOVA, with significant main effects and interactions further analyzed with two-way, sex × time MM-ANOVAs, with session and time as within-subject factors and sex as the between-subject factor. Significant interactions were followed up using Bonferroni’s multiple comparisons post hoc test by comparing each 1-min bin intake between males and females.

To assess front-loading behavior, the average alcohol intake per minute (“average rate of intake”, g/kg/min) calculated from the total alcohol intake data (g/kg) was used as a threshold to compare to the alcohol intake data (g/kg) in 1-min bins. This was analyzed separately for each sex and session using multiple two-way MM-ANOVAs, with time × alcohol intake (intake in 1-min bins versus rate of intake) as within-subject factors. Significant interactions were followed up using Bonferroni’s multiple comparisons post hoc test by comparing each 1-min bin to the average rate of intake.

Access compression was analyzed by three-way, sex × access compression × time and access compression × session × time, with significant main effects and interactions further analyzed using two-way, access compression × time and sex × time MM-ANOVAs, with access compression and time as within-subject factors and sex as the between-subject factor. Bonferroni’s multiple comparisons test was conducted as post hoc analysis following significant main effects or interactions, by comparing the total intake in each 1-min bin between the normal access and the compressed access sessions. To determine whether total alcohol consumed in the first 5-min differed by sex, we used unpaired two-tailed *t*-tests. The accepted level of significance for all tests was *p* ≤ 0.05 and was indicated by different symbols (summarized in Table 1). All significant Bonferroni’s multiple comparisons post hoc tests are indicated by the exact multiplicity adjusted *p* value or as “adjusted *p*s < 0.05” if the multiplicity adjusted *p* values for each comparison is below the set threshold at alpha = 0.05 for the familywise significance level to account for multiple comparison testing [72].

**Table 1:** Overview of statistical indicators used in the figures

Symbol	Significant effect	Statistical analysis	Used in
*	Sex differences	Bonferroni’s multiple comparisons post hoc test	Fig. 2B, inset of Fig. 2E
#	Effect of session	Bonferroni’s multiple comparisons post hoc test	Figs. 2B, 3B
O	Front-loading	Bonferroni’s multiple comparisons post hoc test	Figs. 2D, E, 3D, E, 4D–G
+	Access compression	Bonferroni’s multiple comparisons post hoc test	Fig. 4B, D–G

**Fig. 2:**
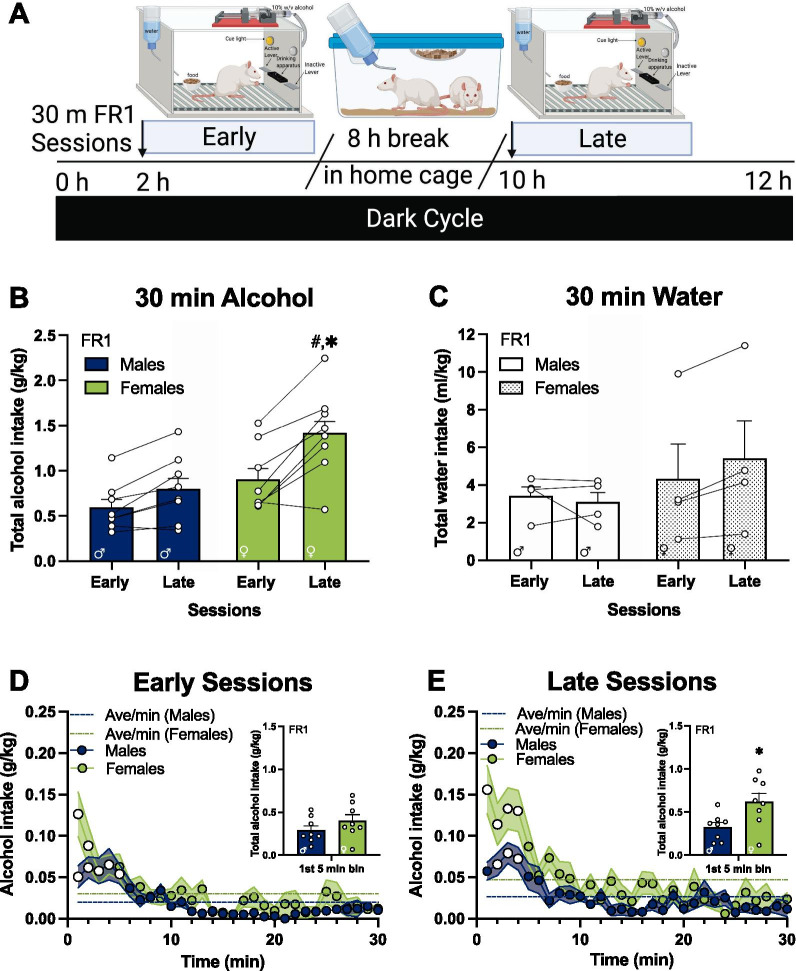
Front-loading behavior drives sex differences in alcohol intake on sessions later in the dark cycle. **A** Rats were placed in operant boxes at 2 h in the dark cycle (“early sessions”) and returned to the home cage after the 30-min session with an FR1 schedule of reinforcement. After 8 h, rats were placed back in the operant boxes at 10 h in the dark cycle (“late sessions”) and returned to the home cage after the 30-min session. **B** Average of total alcohol intake (g/kg) of early and late sessions (n = 8 / sex); **C** average of total water intake (ml/kg) in the control group (n = 4 / sex); **D** early sessions and **E** late sessions of total alcohol intake per minute in the session, inset: total alcohol intake in the first 5-min bin. *FR* fixed ratio, *h* hour, *min* minutes. **B** #, higher intake in late sessions compared to early sessions in females, adjusted *p* < 0.05, and *, higher intake in females compared to males in the late sessions, adjusted *p* < 0.05; both indicated by Bonferroni’s multiple comparisons test post hoc analysis following a session × sex interaction. **D**,** E** White circles, higher intake compared to the average rate of intake of the session in males and females, adjusted *ps* < 0.05; indicated by Bonferroni’s multiple comparisons test post hoc analysis following a time × alcohol intake interaction. In inset graph of **E** *, unpaired *t*-test, *p* < 0.05. Error bars and shading are shown in SEM. **A** is created with BioRender.com

**Fig. 3:**
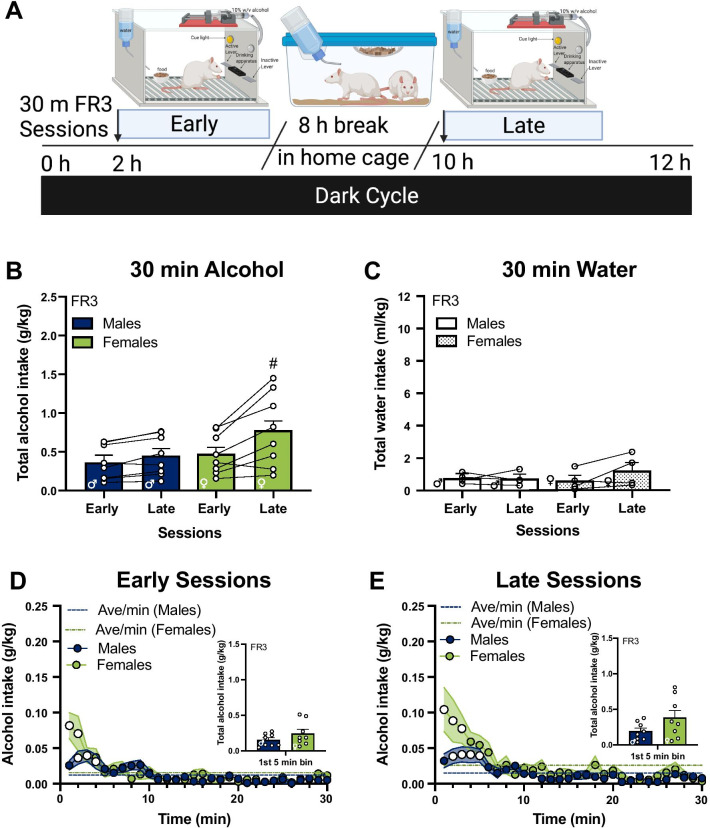
Females maintain front-loading behavior after increasing effort in the late sessions despite decreased total intake. **A** Rats were placed in operant boxes at 2 h in the dark cycle (“early sessions”) and returned to the home cage after the 30-min session with an FR3 schedule of reinforcement. After 8 h, rats were placed back in the operant boxes at 10 h in the dark cycle (“late sessions”) and returned to the home cage after the 30-min session. **B** Average of total alcohol intake (g/kg) of early and late sessions (n = 8 / sex); **C** average of total water intake (ml/kg) in the control group (n = 4 / sex); **D** early sessions and **E** late sessions of total alcohol intake per minute in the session, inset: total alcohol intake in the first 5-min bin. *FR* fixed ratio, *h* hour, *min* minutes. **B** #, higher intake in the late sessions compared to the early sessions in females, adjusted *p* < 0.01; indicated by Bonferroni’s multiple comparisons test post hoc analysis following a session × sex interaction. **D**, **E** White circles, higher intake compared to the average rate of intake of the session in males and females, adjusted *ps* < 0.05; indicated by Bonferroni’s multiple comparisons test post hoc analysis following a time × alcohol intake interaction. Error bars and shading are shown in SEM. **A** is created with BioRender.com

**Fig. 4:**
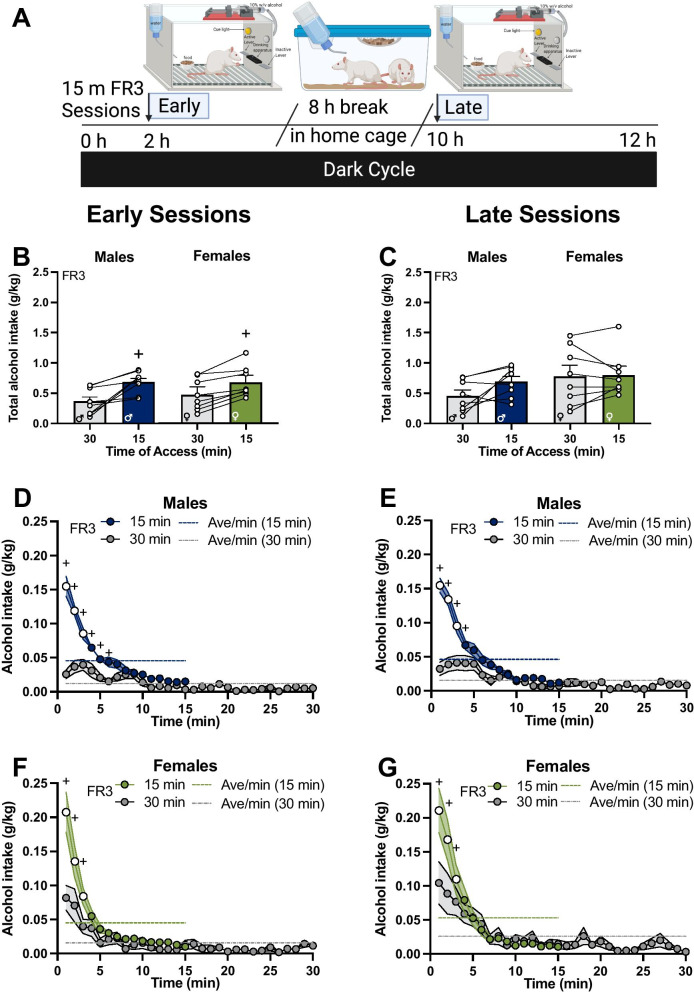
Access time compression increased front-loading of alcohol intake of males to the level of females. **A** Rats were placed in operant boxes at 2 h in the dark cycle (“early sessions”) and returned to the home cage after the 15-min session with an FR3 schedule of reinforcement. After 8 h, rats were placed back in the operant boxes at 10 h in the dark cycle (“late sessions”) and returned to the home cage after the 15-min session. **B** Average of total alcohol intake of early and **C** late sessions (n = 8 / sex); **D** early sessions and **E** late sessions of total alcohol intake per minute in the session of males; **F** early sessions and **G** late sessions of total alcohol intake per minute in the session of females. *FR* fixed ratio, *h* hour, *min* minutes. **B + **, higher intake in the compressed happy hour access (15-min) sessions compared to the normal (30-min) sessions in both males and females, adjusted *p* < 0.05; indicated by a main effect of access compression. **D**–**G) + **, higher intake in the compressed happy hour access (15-min) sessions compared to the normal (30-min) sessions in males (**D** and **E**) and females (**F** and **G**), adjusted *p*s < 0.05; indicated by Bonferroni’s multiple comparisons test post hoc analysis following time × access compression interaction. **D**–**G)** White circles, higher intake compared to the average rate of intake of the session in males and females, adjusted *p*s < 0.05; indicated by Bonferroni’s multiple comparisons test post hoc analysis following time × alcohol intake interaction. Error bars and shading are shown in SEM. **A** is created with BioRender.com

## Results

### Timing of access to alcohol influences active lever responding in males and females

Male rats are capable of reaching identical excessive drinking twice a day when access to alcohol is provided twice in the light cycle with a break between sessions [35]. However, we found significant differences in the total number of active lever presses per minute between sessions early in the dark cycle and sessions late in the dark cycle in all conditions in the alcohol group (main effect of session, *F*_(1, 14)_ = 35.47, *p* < 0.0001 in 30-min FR1 sessions; *F*_(1, 14)_ = 19.69, *p* < 0.001 in 30-min FR3 sessions; and *F*_(1, 14)_ = 5.52, *p* < 0.05 in 15-min FR3 sessions; data not shown). Therefore, we separated the data by session to quantify the alcohol intake difference between the “early sessions” and “late sessions” in male and female rats. Additionally, in the 15-min FR3 sessions we found a main effect of sex (*F*_(1, 14)_ = 7.86, *p* < 0.05) and thus, for the access compression analysis we separated the data by sex as well. This was not found in the total number of active lever presses per minute of control rats that only had access to water (effect of session, *F*_(1, 6)_ = 0.44, *p* > 0.05 in 30-min FR1 sessions; effect of session, *F*_(1, 6)_ = 1.42, *p* > 0.05 in 30-min FR3 sessions; effect of session, *F*_(1, 6)_ = 1.03, *p* > 0.05 in 15-min FR3 sessions; data not shown). There was a session x sex interaction (*F*_(1, 6)_ = 6.23, *p* < 0.05 in 15-min FR3 sessions; data not shown), however, post hoc analysis did not show any significance in either sex or session (Bonferroni’s multiple comparisons test, adjusted *p* > 0.05).

### Sex differences in voluntary alcohol intake is greatest when testing occurs later in the dark cycle

Operant self-administration experiments in rodents allows for the precise study of alcohol drinking patterns and examine the role of sex in alcohol consumption behavior [49–56]; reviewed in [57, 58]. In the current study design (see details in Fig. 2A), the average of total active lever presses within the 30-min sessions were not different between males and females (effect of sex, *F*_(1, 14)_ = 0.13, *p* > 0.05; data not shown), yet the average of total alcohol intake (g/kg) was higher in adult female rats compared to males (Fig. 2B; main effect of sex, *F*_(1, 14)_ = 6.67, *p* < 0.05). Alcohol intake was higher in the late sessions compared to the early sessions (main effect of session, *F*_(1,14)_ = 34.97, *p* < 0.0001). There was also a session × sex interaction (*F*_(1,14)_ = 6.34, *p* < 0.05) and post hoc analysis found significantly higher alcohol intake in females compared to males only in the late sessions (Fig. 2B; Bonferroni’s multiple comparisons test, adjusted *p* < 0.01), but not in the early sessions (adjusted *p* > 0.05*)*. Rats consumed significantly more alcohol in the late sessions compared to the early sessions within females (Fig. 2B; Bonferroni’s multiple comparisons test, adjusted *p* < 0.0001), whereas within males, this was only a trend (adjusted *p* = 0.06). Higher alcohol intake in the later sessions was not due to non-discriminative lever pressing as inactive lever presses were comparable between the early and late sessions (*F*_(1, 14)_ = 0.01, *p* > 0.05; Table 2). In control rats, water intake (ml/kg) did not differ between males and females (Fig. 2C; *F*_(1, 6)_ = 0.61, *p* > 0.05), or between the early and late sessions (*F*_(1, 6)_ = 1.35, *p* > 0.05).

**Table 2:** Effect of sex and session on inactive lever presses

Operant parameters	Inactive lever (presses/minute) Mean (± *SEM*)			MM ANOVA: sex × session			
	Session	Males	Females	Effect	*p-*value	*F*	*Df*
30-min FR1 sessions	Early	0.1900 (*0.04*)	0.1442 (*0.03*)	Sex	0.9228	0.01	1, 14
	Late	0.1450 (*0.04*)	0.1825 (*0.03*)	Session	0.9043	0.01	
				Sex × session	0.1483	2.34	
30-min FR3 sessions	Early	0.0792 (*0.03*)	0.0842 (*0.02*)	Sex	0.2596	1.38	1, 14
	Late	0.0825 (*0.02*)	0.1625 (*0.04*)	Session	0.0558	4.35	
				Sex × session	0.0761	3.67	
15-min FR3 sessions	Early	0.1463 (*0.07*)	0.1218 (*0.02*)	Sex	0.6515	0.21	1, 14
	Late	0.2199 (*0.07*)	0.1783 (*0.02*)	Session	0.0097^#^	8.96	
				Sex × session	0.6989	0.16	

*MM-ANOVA*  repeated measures analysis of variance

^#^*p* < 0.05

### Females drink faster and consume more alcohol than males later in the dark cycle

We followed with a microstructural analysis of alcohol drinking, measuring the total alcohol intake per 1-min bins across the operant session. In the early sessions, time had a significant effect on alcohol intake (Fig. 2D; main effect of time, *F*_(29, 406)_ = 10.40, *p* < 0.0001) but there were no significant differences between males and females (*F*_(1,14)_ = 3.81, *p* = 0.07). Post hoc analyses found sex differences only in the first minute of the session (Bonferroni’s multiple comparisons test, adjusted *p* < 0.0001). In contrast, in the late sessions there is a main effect of time (Fig. 2E; *F*_(29, 406)_ = 11.86, *p* < 0.0001), a main effect of sex (*F*_(1, 14)_ = 8.52, *p* < 0.05) and a time × sex interaction (*F*_(29, 406)_ = 2.07, *p* < 0.01). Post hoc analyses found sex differences in the minute 1 and minute 4 of the session (Bonferroni’s multiple comparisons test, adjusted *p*s < 0.05), with females drinking more alcohol than males.

For an accurate front-loading behavior analysis, total alcohol intake per 1-min bins was compared to the average rate of intake. In the sessions early in the dark cycle, there was a significant time × alcohol intake interaction (*F*_(29, 406)_ = 7.66, *p* < 0.0001) in males, with follow-up post hoc analyses found significantly higher alcohol intake in the first 5 min of the session (white circles, Fig. 2D; Bonferroni’s multiple comparisons test, adjusted *ps* < 0.05) accounting for an average of 48% of the total alcohol intake at the start of access which is characteristic of front-loading [43]. A significant time × alcohol intake interaction was also found in females (*F*_(29, 406)_ = 5.20, *p* < 0.0001), with post hoc analyses finding significantly higher intake in the first 2 min (white circles, Fig. 2D; Bonferroni’s multiple comparisons test, adjusted *ps* < 0.05), which accounts for 32% of the total alcohol intake.

After an 8-h break in the home cage, rats were placed in the operant box for the sessions later in the dark cycle. Front-loading analysis of the alcohol intake in 1-min bins in the late sessions of males found a significant time × alcohol intake interaction (*F*_(29, 406)_ = 5.98, *p* < 0.0001). Follow-up post hoc analyses found that males showed significantly higher intake on minute 2, 3 and 4 of the session (white circles, Fig. 2E; Bonferroni’s multiple comparisons test, adjusted *p*s < 0.05) which accounts for 29% of the total intake in the late sessions. In females, a significant time × alcohol intake interaction was also found in the late sessions (*F*_(29, 406)_ = 7.29, *p* < 0.0001), with follow-up post hoc analyses finding significantly higher intake in the first 4 min of the session (white circles, Fig. 2E; Bonferroni’s multiple comparisons test, adjusted *p*s < 0.05) accounting for an average of 37% of the total intake within females.

The total amount of alcohol ingested during the first 5 min was significantly higher in females compared to males in the late sessions (Fig. 2E inset; unpaired *t*-test, *p* < 0.05), but no sex differences were found in the early sessions (Fig. 2D inset; unpaired *t*-test, *p* > 0.05).

### Increasing the effort required to access alcohol reduces total alcohol intake

To test if increasing the effort to obtain alcohol reduces drinking to the same degree in both males and females, we manipulated the fixed ratio schedule of deliveries from FR1 to FR3, i.e., shifting from requiring 1 to requiring 3 presses for alcohol delivery (Fig. 3A). This increase in effort suppressed the total amount of alcohol self-administered during the 30-min session in both male and female rats (main effect of effort, *F*_(1, 14)_ = 29.55, *p* < 0.0001 in early sessions and *F*_(1, 14)_ = 31.10, *p* < 0.0001 in late sessions, for comparisons to FR1, see Fig. 2), resulting in a loss of sex difference in total alcohol intake (Fig. 3B; *F*_(1, 14)_ = 2.04, *p* > 0.05). There was a main effect of session (Fig. 3B; *F*_(1, 14)_ = 16.82, *p* < 0.01) and a session × sex interaction (Fig. 3B; *F*_(1,14)_ = 5.28, *p* < 0.05). Post hoc analysis found higher alcohol consumption in the late sessions compared to the early sessions within females (Bonferroni’s multiple comparisons test, adjusted *p* < 0.001), but not within males.

In the FR3 sessions, control rats continue to show a lack of sex differences in self-administration of water (Fig. 3C; *F*_(1,6)_ = 0.17, *p* > 0.05). There was also no effect of time of session on self-administration of water (Fig. 3C; *F*_(1,6)_ = 1.84, *p* > 0.05). There was a trend for higher responding on the inactive lever in the sessions later in the dark cycle, but this was not significant (*F*_(1, 14)_ = 4.35, *p* = 0.06; shown in Table 2), along with a non-significant trend for a session × sex interaction (*F*_(1, 14)_ = 3.67, *p* = 0.08; shown in Table 2).

### Higher front-loading drinking behavior persists in females even when higher effort is required for access to alcohol

We next tested whether sex differences in the microstructural analysis of drinking persisted when rats had to work harder for alcohol. In the early sessions, there was a main effect of time (Fig. 3D; *F*_(29, 406)_ = 10.66, *p* < 0.0001) and time × sex interaction (*F*_(29, 406)_ = 2.34, *p* = 0.0002). Further, post hoc analyses found higher drinking in females in the first 2 min of the session compared to males (Bonferroni’s multiple comparisons test, adjusted *p*s < 0.05). In the late sessions there was a main effect of time (Fig. 3E; *F*_(29, 406)_ = 10.58, *p* < 0.0001) and time × sex interaction (*F*_(29, 406)_ = 2.18, *p* = 0.0005), and similar to the early sessions, post hoc analyses found that females showed higher drinking in the first 2 min of the session compared to males (Bonferroni’s multiple comparisons test, adjusted *p*s < 0.05).

Analysis of front-loading behavior comparing alcohol intake in 1-min bins to the average rate of intake in males found a significant time × alcohol intake interaction (*F*_(29, 406)_ = 7.15, *p* < 0.0001) in the early sessions, with follow-up post hoc analyses showing higher intake from minute 2 to minute 4 of the session (white circles, Fig. 3D; Bonferroni’s multiple comparison’s test, adjusted *p*s < 0.05). In females, a significant time × alcohol intake interaction was found in the early sessions (*F*_(29, 406)_ = 6.30, *p* < 0.0001), with follow-up post hoc analyses showing significantly higher intake in the first 2 min of the session (white circles, Fig. 3D; Bonferroni’s multiple comparison’s test, adjusted *p*s < 0.05). In the early sessions, 31% of the total alcohol intake occurs from minute 2 to minute 4 of the session in males while the same (31%) occurs in the first 2 min of the session in females.

Similar to the early sessions, front-loading analysis of males in the late sessions found a time × alcohol intake interaction (*F*_(29, 406)_ = 6.38, *p* < 0.0001), with follow-up post hoc analyses showing higher intake in males from minute 2 to minute 5 of the session (white circles, Fig. 3E; Bonferroni’s multiple comparison’s test, adjusted *p*s < 0.05). Within females in the late sessions, front-loading analysis found a time × alcohol intake interaction (*F*_(29, 406)_ = 6.39, *p* < 0.0001). Post hoc analyses found higher alcohol intake in the first 3 min of the session compared to the average rate of intake in females (white circles, Fig. 3E; Bonferroni’s multiple comparison’s test, adjusted *p*s < 0.05). Approximately 36% of intake occurs from minute 2 to minute 5 of the session in males while 34% occurs in the first 3 min of the session in females in the late sessions. This suggests that under FR3 conditions, females still exhibit more front-loading behavior compared to males, especially later in the dark cycle. However, despite higher levels of front-loading in females, the total amount of alcohol consumed in the first 5 min of the session was no longer significantly different between males and females in the early or late sessions (Fig. 3D inset, early sessions, and Fig. 3E inset, late sessions; unpaired *t*-tests, *p* > 0.05).

### Alcohol drinking levels were not further increased by testing every other day (intermittent schedule)

Intermittent access to alcohol can augment alcohol intake in voluntary rodent models [68, 69]. We investigated whether a shift in the schedule (testing every other day rather than consecutive days) would enhance drinking levels. Analysis of two different schedules of alcohol access during the week (intermittent access versus continuous access) indicated there were no measurable differences in total alcohol intake over the session (*F*_(1, 14)_ = 0.23, *p* > 0.05; data not shown) or in microstructural analysis per 1-min intake (*F*_(1, 14)_ = 0.06, *p* > 0.05; data not shown). Based on these results, we pooled the data from both testing schedules in our analyses of the 15-min FR3 (happy hour) sessions. There was one day in which the session early in the dark cycle could not be performed due to loss of power; thus, we excluded those data from the analyses.

### Compressing access time promotes faster drinking and elevates total intake

To determine whether a happy hour model would promote higher levels of front-loading behavior and alcohol consumption in males and females, we reduced testing sessions from 30 to 15 min (“access compression”, Fig. 4A). Limiting alcohol availability elicited higher total alcohol intake in the early sessions (Fig. 4B; main effect of access compression, *F*_(1, 14)_ = 33.93, *p* < 0.0001) with the happy hour sessions eliciting higher total intake compared to the normal sessions in both sexes. In the late sessions, access compression had no significant effect on the total intake (Fig. 4C; *F*_(1, 14)_ = 3.16, *p* = 0.09).

To gain insight into the specific patterns of drinking that lead to higher total intake in the compressed sessions, we compared alcohol intake in 1-min bins of the compressed 15-min happy hour sessions to the normal 30-min sessions. We found that compression of access time increased front-loading of males in the early sessions (Fig. 4D; time × access compression interaction, *F*_(14, 98)_ = 24.94, *p* < 0.0001) and late sessions (Fig. 4E; time × access compression interaction, *F*_(14, 98)_ = 34.63, *p* < 0.0001). In females, compression of access time also increased front-loading in the early sessions (Fig. 4F; time × access compression interaction, *F*_(14, 98)_ = 13.47, *p* < 0.0001), and in the late sessions (Fig. 4G; time × access compression interaction, *F*_(14, 98)_ = 15.77, *p* < 0.0001). Post hoc analyses in males found significantly higher alcohol intake after access compression in the first 6 min of the early sessions and the first 4 min of the late sessions compared to the normal sessions (+ s in Fig. 4D and E, Bonferroni’s multiple comparisons test, adjusted *p*s < 0.05). In females, post hoc analyses found significantly higher alcohol intake in the first 3 min in both early and late sessions of the happy hour sessions compared to the normal sessions (**+ **s in Fig. 4F and G, Bonferroni’s multiple comparisons test, adjusted *p*s < 0.05). As such, the data suggests that limiting the time of alcohol availability elicited higher front-loading drinking behavior in males and females in both sessions in the dark cycle, and this rapid rate of drinking resulted in more total alcohol intake overall in the happy hour sessions compared to the normal sessions.

### Compressing access time causes males to engage in rapid and heavier alcohol intake that is comparable to females

**Fig. 5:**
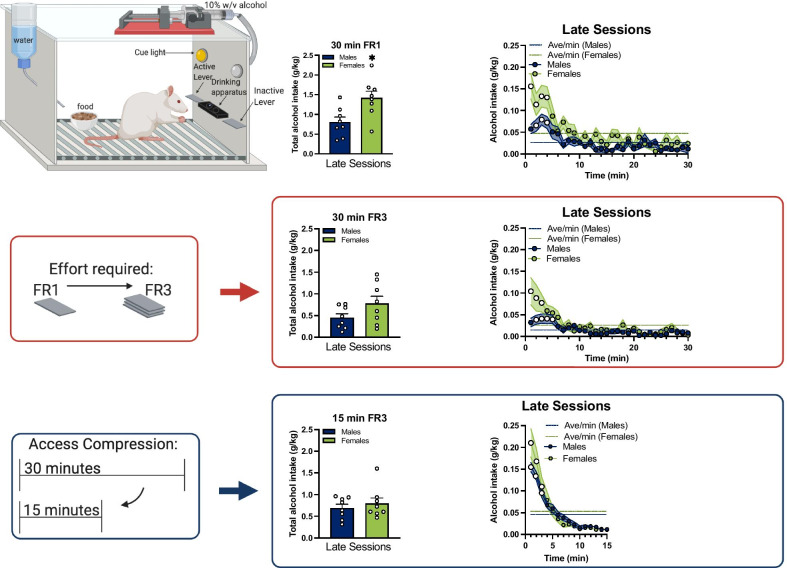
Summary of findings. All graphs show alcohol intake (g/kg) in the late sessions for all conditions. *w/v* weight per volume, *FR* fixed ratio. Error bars and shading are shown in SEM. Created with BioRender.com

Between males and females, alcohol intake in 15 min of access showed a significant effect of time (*F*_(14, 196)_ = 77.90, *p* < 0.0001) and time × sex interaction (*F*_(14, 196)_ = 2.33, *p* < 0.01) in the early sessions, as well as in the late sessions (effect of time, (*F*_(14, 196)_ = 72.22, *p* < 0.0001); time × sex interaction, *F*_(14, 196)_ = 2.20, *p* < 0.01). Post hoc analyses found that sex differences in drinking were only found in the first minute of the access in both early and late sessions (Bonferroni’s multiple comparisons test, adjusted *p*s < 0.05; these subtle sex differences can be viewed visually comparing the first minute in Fig. 4C versus Fig. 4E and Fig. 4D versus Fig. 4F and in Fig. 5).

Front-loading analyses found a time × alcohol intake interaction in both early and late sessions in males (*F*_(14, 196)_ = 66.31, *p* < 0.0001 in early sessions; *F*_(14, 196)_ = 97.04, *p* < 0.0001 in late sessions) and females (*F*_(14, 196)_ = 32.84, *p* < 0.0001 in early sessions; *F*_(14, 196)_ = 28.10, *p* < 0.0001 in late sessions). Furthermore, post hoc analyses indicated that both males and females consume significantly more alcohol in the first 3 min of the session compared to the average rate of intake (white circles in Fig. 4D–G and in Fig. 5; Bonferroni’s multiple comparisons test, adjusted *p*s < 0.05) in both early and late sessions. In males, alcohol intake during front-loading accounts for 55% of the total intake in the early sessions and 60% in the late sessions. In females, alcohol intake during front-loading accounts for 63% of the total intake in the early sessions and 61% in the late sessions. Thus, access compression drove up front-loading behavior to such an extent in males that alcohol drinking patterns were almost indistinguishable from drinking patterns in females.

Control rats showed no differences in water intake between males and females (*F*_(1, 6)_ = 1.72, *p* > 0.05) nor differences between time of the session (*F*_(1, 6)_ = 0.05, *p* > 0.05, data not shown). Inactive lever pressing per minute was found higher in the late sessions compared to the early sessions (main effect of session, *F*_(1, 14)_ = 8.96, *p* < 0.01; shown in Table 2), with post hoc analyses finding only a trend in males (Bonferroni’s multiple comparisons test, adjusted *p* = 0.06) but not females (adjusted *p* > 0.05). A significant main effect of session was found in the active lever presses per minute (*F*_(1, 14)_ = 5.52, *p* < 0.05; data not shown) and a main effect of sex (*F*_(1,14)_ = 7.8, *p* < 0.05; data not shown). However, post hoc analysis found no significant differences in the active lever presses per minute between the sessions in males (Bonferroni’s multiple comparisons test, adjusted *p* > 0.05; data not shown) and a significant difference in females (adjusted *p* = 0.05; data not shown).

## Discussion

### Main findings

The factors motivating drinking and the health consequences of heavy alcohol use can differ with sex [29]. Adolescent girls report drinking alcohol as a coping mechanism to alleviate depression and psychological distress because of its acute anxiety-reducing properties, while drinking in adolescent boys tends to relate more to risk-taking and impulsive behaviors [73, 74]. Additionally, in adulthood women are more likely to alter their drinking behavior (drinking more, drinking more quickly, or both) in response to incentives that promote drinking, e.g., “happy hour” [34]. Women are reported to have more detrimental emotional and physical consequences including emotional instability, depression, and anxiety compared to men who engage in binge drinking [30, 75]. There are several factors that could contribute to a higher prevalence of mental health disorders associated with alcohol use in women. For example, women of varying races, ages, and nationalities are more likely than men to report mental health struggles and seek treatment due to stigma that negatively affects men more than women [76]. As another result of stigmatization of mental health, medical doctors are more likely to diagnose depression in women than men, even with comparable standardized scores for depression or with identical symptoms [77]. In addition to these social factors, there could also be subtle differences in drinking patterns between men and women that could influence the severity of negative consequences of alcohol on the brain. Questions about the sensitivity of the brain to equivalent doses of alcohol can be answered using rodent models, as rats and mice with a chronic history of alcohol and withdrawal cycles exhibit affective disturbances similar to alcohol used disorder in humans [78–83]. Nevertheless, it can be challenging to test the effect of alcohol drinking on the nervous system in rodents because adult female rodents tend to drink significantly more g/kg alcohol than their male counterparts [42, 59–63, 84, 85]. Studies that show no sex differences in alcohol consumption can also be found, which may reflect differences in species, strain, age, drinking paradigm, and study design [46, 47, 63, 65, 86–88]. While it is well known in the field that adult female rats drink more alcohol than males, the specific differences in patterns of drinking between males and females and their effects on the brain are not well understood. Herein, we used operant procedures to characterize the temporal dynamics of alcohol drinking in adult male and female rats to identify the specific pattern of drinking that drives the sex differences in intake and tested how changes in alcohol access (happy hour) can influence these sex differences. Collectively, we found that higher intake in females occurs during the initiation of access, i.e., front-loading. Moreover, manipulation of operant conditions such as access time to model happy hour resulted in both front-loading behavior and total alcohol intake in males being elevated to the level of females; for a summary of the results see Fig. 5. This study overall provides insight into sex differences in drinking behavior and provides a novel approach to control voluntary intake and study its effects on the brain.

### Front-loading behavior causes higher alcohol intake in females

Consistent with previous reports, female rats drank more alcohol than males in the current study [59–63, 85, 87]; reviewed in [29]. The sex difference was most robust when rats were tested late in the dark cycle and effort was minimal (FR1 schedule of reinforcement). While males and females both showed “front loading” drinking behavior, the amount of alcohol consumed during this time was much higher in females. Females drank almost as much alcohol in the first 5 min as males drank in the entire 30 min session. It is important to note that g/kg alcohol intake is calculated based on the number of lever presses. Although our approach is less precise than the lickometer method [38, 40, 42–44, 53, 89], we always confirm there is no liquid left in the drinking apparatus or evidence of spillover in the bedding after each session. Thus, we have found lever presses to be a reliable index of alcohol intake, and this is further supported by previous reports in which we used indwelling catheters to measure blood alcohol levels at multiple time points during operant self-administration [90, 91].

Front-loading behavior is presumed to reflect the level of motivation to experience the rewarding effects of alcohol. Consistent with this interpretation, front-loading is observed in high drinking mouse strains [42–48], in alcohol-dependent rats [39], and in models that use chronic intermittent alcohol access to induce compulsive-like alcohol drinking in rats [40]. High levels of front-loading has been observed in other experimental models in which there is a motivation to seek a specific substance, e.g., following intermittent access to high-fat sweetened liquid in rats [38], with sodium depletion in mice [37] and polycose in rats [36].

Drinking alcohol quickly may be increasing the probability of intoxication in females. The intoxicating effects of alcohol depend not only on the speed at which alcohol is ingested, but also on the speed at which alcohol is metabolized and cleared from the body. Faster metabolism has been reported in female rodents compared to males, with mixed findings reported in humans [92–101]; reviewed in [16, 102, 103]. Thus, one possibility is that females drink alcohol more quickly because they metabolize it faster. However, front-loading behavior could be altered in our study by changing operant parameters that are unlikely to affect metabolic processes. Increasing the effort to obtain alcohol with an FR3 schedule suppressed front-loading. Conversely, compressing access time (modeling happy hour) had the opposite effect on front-loading. These experimental manipulations may serve as useful strategies to explore the mechanisms underlying drinking behavior.

### Manipulation of operant parameters as a strategy to study sex differences

As mentioned above, the current study outlines an approach in which manipulation of operant parameters can serve to circumvent sex differences in drinking in adult rats. This strategy would be useful for studies investigating the effects of alcohol intake on the brain. Involuntary/forced models of alcohol administration are commonly used because they allow for delivery of equal amounts of low, moderate, and high levels of alcohol in males and females, albeit with different degrees of aversive consequences; for a review of current preclinical models of alcohol drinking see [57]. With voluntary drinking models, operant parameters can be manipulated to bring one group’s intake up or down to the level of another group. For example, operant lever access can be controlled to limit the maximum number of rewards to match glucose intake in control groups consuming sweetened water to the glucose intake in groups consuming sweetened alcohol [64, 65, 104]. Additionally, it is possible to decrease intake of female mice to the level of males by changing the alcohol concentration available for drinking from 15 to 10% v/v [105]. Conceptionally this same strategy could be used to reduce alcohol intake in adult females to the level of adult males, but this limits experimental design to testing only lower levels of voluntary alcohol. In the current study, we found that drinking was reduced, producing similar total intake between males and females, after increasing the effort required to obtain alcohol (a shift from an FR1 to an FR3 schedule of reinforcement) which was sufficient to dampen sex differences. However, the increase in effort did not reduce front-loading of females to the level of males, as sex differences in front-loading remained. Our study replicated an earlier report in male rats [35], and extended these findings by demonstrating that compressing the time of access to model a happy hour incentivized rats of both sexes to drink more alcohol, more rapidly, and that its effects were more pronounced in male rats. This resulted in higher alcohol intake in males, eliciting female-like drinking patterns; thus, this approach can increase the opportunities for testing the effects of voluntary alcohol on neural or behavioral measures. Furthermore, this rodent model of voluntary alcohol drinking can be used to study the neuronal activity underlying front-loading behavior, which ultimately may help develop targeted treatment strategies for alcohol misuse and AUD.

### Effect of time of day on alcohol intake

Alcohol drinking tests in rodents are often conducted within the first few hours of the dark cycle to take advantage of increased nocturnal activity [84, 106–109]; reviewed in [110]. In the current study, we show that alcohol consumption within females was highest when testing was done 10 h into the dark cycle. Higher intake in the later testing sessions is not likely an effect of non-discriminative lever pressing because inactive lever presses did not differ between early and late sessions in females. Instead, other factors must underlie higher drinking in females when testing is done later in the dark cycle. One possibility is that early sessions may have acted as a primer for increased alcohol drinking in the late sessions, as all rats were tested twice daily in this within-subject design. To explore this further, we used a data set from a day in which rats only had a late session due to a power outage earlier in the day, which had to be excluded from the overall statistical analysis. We compared the drinking behavior of that day to a single late session from two days before, in which rats had an early session as well and found no differences in alcohol intake. This suggests that those early sessions are not necessary for driving higher levels of intake in the late sessions. Of course, more data would be needed to exclude this explanation completely. Circadian variations in biological factors such as stress-related hormones may contribute to higher drinking in the later sessions. For example, baseline plasma corticosterone levels in rodents have a temporally regulated rhythm, with higher levels peaking around two hours before the start of the dark cycle and gradually decreasing until reaching low levels around 10 h into the dark cycle in both males and females [111, 112]. Low levels of corticosterone in rodents and cortisol in humans is associated with higher drinking [75, 90, 113], reviewed in [114]. If the circadian reduction in corticosterone levels late in the dark cycle were the main factor driving higher drinking levels at that time, we might expect that drinking in females would be highest when baseline corticosterone levels were lowest (late in the dark cycle, as tested here or early in the light cycle, which was not tested here). While that prediction is consistent with our data in females, a thorough analysis of drinking behavior at various timepoints across the light and dark cycle of male and female rats would be necessary to answer this question. Moreover, low corticosterone levels cannot explain sex differences because female rats actually have higher corticosterone levels than males [112, 115]. Future studies could help determine whether circadian regulation play a role in sex differences in alcohol intake and how this corresponds with stress-related hormones such as corticosterone.

### Blood alcohol levels

Herein, most of the alcohol drinking occurred in the first 5 min of access, regardless of the total length of the session (30 versus 15 min). Based on our current findings on front-loading, this suggests that collection of blood samples should take place after a few minutes of initial alcohol access for accuracy in binge drinking rodent models. Yet, blood alcohol concentration levels are usually taken after testing typically around 30 min to 1 h after the first initial intake [116]. When blood alcohol levels were assessed at an earlier timepoint during alcohol during a 2-h DID drinking session (5–12 min), these levels were more aligned with drinking behavior compared to levels assessed near the end of the session (112–119 min) [43]. Blood alcohol levels were not measured in our study; however, based on previous studies in male rats, the total consumption of 1.25 g/kg of alcohol or more reaches the blood alcohol levels of 0.08 g/dL or higher [64]. Other studies using the DID paradigm in mice show that adult females consume more alcohol and have higher blood alcohol levels compared to males [42, 106, 107, 117]. Mice metabolize alcohol faster than rats and humans [57]; thus, taking blood samples too long after the initial drinking for assessing blood alcohol levels may result in inaccurate readouts. An important goal for future studies is to assess more accurate blood alcohol levels early in the operant session in both male and female rats while also taking into account the detrimental effects various alcohol metabolites may cause to cells and circuits in the brain [118]; reviewed in [13, 119].

### Perspectives and significance

There is a human health discrepancy between the prevalence of alcohol drinking and its mental health consequences in men and women. To understand this more, we need to get a better sense of how drinking patterns may differ with sex. Our study shows that female rats drink excessively at the start of a drinking session (front-loading). If the same patterns occur in women, it may have implications for how alcohol affects the brain and the body. We also found that we can manipulate operant parameters to increase front-loading in male rats to drive up alcohol intake to the level of females. This means that we can now take advantage of these manipulations to directly compare effects of alcohol drinking on the male and female brain. This model can help us in the future have a more precise understanding of the neuronal activity underlying these patterns of drinking behavior which may play a role in the detrimental effects on mental health that differ by sex.

## Conclusion

Understanding the underlying patterns of alcohol drinking behaviors that drive sex differences may help in determining factors contributing to AUD vulnerability. Overall, we found that manipulation of operant parameters of alcohol availability altered behavioral patterns of drinking in both male and female rats. This manipulation can be used to effectively study sex differences in alcohol-driven effects on the brain. Front-loading behavior is highest 10 h into the dark cycle in females which drives the sex differences in the total alcohol intake. However, under happy hour conditions, adult male rats drink as much alcohol as females. Ultimately, the time of access to alcohol, the effort required to obtain it, and the time of day of testing all contribute to changes in front-loading behavior and increasing or decreasing the magnitude of sex differences in voluntary alcohol intake in adult rats.

## Data Availability

The datasets used and/or analyzed during the current study are available from the corresponding author on reasonable request.

## References

[1] White AM, Slater ME, Ng G, Hingson R, Breslow R (2018). Trends in alcohol-related emergency department visits in the United States: results from the nationwide emergency department sample, 2006 to 2014. Alcohol Clin Exp Res.

[2] American Psychiatric Association. Diagnostic and statistical manual of mental disorders. 5th edn. Online. Arlinton, VA. 2013; doi: 10.1176/appi.books.9780890425596.

[3] Grant BF, Goldstein RB, Saha TD, Chou SP, Jung J, Zhang H (2015). Epidemiology of DSM-5 alcohol use disorder. JAMA Psychiat.

[4] Grant BF, Chou SP, Saha TD, Pickering RP, Kerridge BT, Ruan WJ (2017). Prevalence of 12-month alcohol use, high-risk drinking, and DSM-IV alcohol use disorder in the United States, 2001–2002 to 2012–2013. JAMA Psychiat.

[5] White AJ, DeRoo LA, Weinberg CR, Sandler DP (2017). Lifetime alcohol intake, binge drinking behaviors, and breast cancer risk. Am J Epidemiol.

[6] Mann KF, Ackermann K, Croissant B, Mundle G, Nakovics H, Diehl A (2005). Neuroimaging of gender differences in alcohol dependence: are women more vulnerable?. Alcohol Clin Exp Res.

[7] Schwarzinger M, Thiébaut SP, Baillot S, Mallet V, Rehm J (2018). Alcohol use disorders and associated chronic disease—a national retrospective cohort study from France. BMC Public Health.

[8] Kerr-Corrêa F, Igami TZ, Hiroce V, Tucci AM (2007). Patterns of alcohol use between genders: a cross-cultural evaluation. J Affect Disord.

[9] Hamajima N, Hirose K, Tajima K, Rohan T, Calle EE, Heath CW (2002). Alcohol, tobacco and breast cancer—collaborative reanalysis of individual data from 53 epidemiological studies, including 58,515 women with breast cancer and 95,067 women without the disease. Br J Cancer.

[10] Loft S, Olesen K-L, Døssing M (1987). Increased susceptibility to liver disease in relation to alcohol consumption in women. Scand J Gastroenterol.

[11] Smith-Warner SA, Spiegelman D, Yaun S-S, van den Brandt PA, Folsom AR, Goldbohm RA (1998). Alcohol and breast cancer in women. JAMA.

[12] Urbano-Márquez A (1995). The greater risk of alcoholic cardiomyopathy and myopathy in women compared with men. JAMA J Am Med Assoc.

[13] Erol A, Karpyak VM (2015). Sex and gender-related differences in alcohol use and its consequences: contemporary knowledge and future research considerations. Drug Alcohol Depend.

[14] Erol A, Ho AMC, Winham SJ, Karpyak VM (2019). Sex hormones in alcohol consumption: a systematic review of evidence. Addict Biol.

[15] McCaul ME, Roach D, Hasin DS, Weisner C, Chang G, Sinha R (2019). Alcohol and women: a brief overview. Alcohol Clin Exp Res.

[16] Mancinelli R, Vitali M, Ceccanti M (2009). Women, alcohol and the environment: an update and perspectives in neuroscience. Funct Neurol.

[17] Salvatore JE, Cho SB, Dick DM (2017). Genes, environments, and sex differences in alcohol research. J Stud Alcohol Drugs.

[18] Fede SJ, Abrahao KP, Cortes CR, Grodin EN, Schwandt ML, George DT (2020). Alcohol effects on globus pallidus connectivity: role of impulsivity and binge drinking. PLoS ONE.

[19] Kvamme TL, Schmidt C, Strelchuk D, Chang-Webb YC, Baek K, Voon V (2016). Sexually dimorphic brain volume interaction in college-aged binge drinkers. NeuroImage Clin.

[20] Mashhoon Y, Czerkawski C, Crowley DJ, Cohen-Gilbert JE, Sneider JT, Silveri MM (2014). Binge alcohol consumption in emerging adults: anterior cingulate cortical “thinness” is associated with alcohol use patterns. Alcohol Clin Exp Res.

[21] National Institute on Alcohol Abuse and Alcoholism. NIAAA council approves definition of binge drinking. NIAAA Newsl. 2004;3:3. http://pubs.niaaa.nih.gov/publications/Newsletter/winter2004/Newsletter_Number3.pdf.

[22] Åberg F, Helenius-Hietala J, Puukka P, Jula A (2017). Binge drinking and the risk of liver events: a population-based cohort study. Liver Int.

[23] Gowin JL, Sloan ME, Stangl BL, Vatsalya V, Ramchandani VA (2017). Vulnerability for alcohol use disorder and rate of alcohol consumption. Am J Psychiatry.

[24] Becker JB, Perry AN, Westenbroek C (2012). Sex differences in the neural mechanisms mediating addiction: a new synthesis and hypothesis. Biol Sex Differ.

[25] George O, Koob GF, Vendruscolo LF (2014). Negative reinforcement via motivational withdrawal is the driving force behind the transition to addiction. Psychopharmacology.

[26] Koob GF (2013). Addiction is a reward deficit and stress surfeit disorder. Front Psychiatry.

[27] Peltier MR, Verplaetse TL, Mineur YS, Petrakis IL, Cosgrove KP, Picciotto MR (2019). Sex differences in stress-related alcohol use. Neurobiol Stress..

[28] Mason BJ (2017). Emerging pharmacotherapies for alcohol use disorder. Neuropharmacology.

[29] Flores-Bonilla A, Richardson HN (2020). Sex differences in the neurobiology of alcohol use disorder. Alcohol Res Curr Rev.

[30] Patrick ME, Terry-McElrath YM, Evans-Polce RJ, Schulenberg JE (2020). Negative alcohol-related consequences experienced by young adults in the past 12 months: differences by college attendance, living situation, binge drinking, and sex. Addict Behav.

[31] Substance Abuse and Mental Health Services Administration. Results from the 2014 national survey on drug use and health (NSDUH): detailed tables. US Dep Heal Hum Serv. 2014;Table 2.46B. https://www.samhsa.gov/data/sites/default/files/NSDUH-DetTabs2014/NSDUH-DetTabs2014.htm#tab2-41b.

[32] Babor TF, Mendelson JH, Greenberg I, Kuehnle J (1978). Experimental analysis of the “happy hour”: effects of purchase price on alcohol consumption. Psychopharmacology.

[33] Kuo M, Wechsler H, Greenberg P, Lee H (2003). The marketing of alcohol to college students: the role of low prices and special promotions. Am J Prev Med.

[34] Baldwin JM, Stogner JM, Miller BL (2014). It’s five o’clock somewhere: an examination of the association between happy hour drinking and negative consequences. Subst Abuse Treat Prev Policy.

[35] Jeanblanc J, Sauton P, Jeanblanc V, Legastelois R, Echeverry-Alzate V, Lebourgeois S (2018). Face validity of a pre-clinical model of operant binge drinking: just a question of speed. Addict Biol.

[36] Davis JD (1996). Microstructural analysis of the ingestive behavior of the rat ingesting polycose. Physiol Behav.

[37] D’Aquila PS, Rossi R, Rizzi A, Galistu A (2012). Possible role of dopamine d1-like and d2-like receptors in behavioural activation and “contingent” reward evaluation in sodium-replete and sodium-depleted rats licking for NaCl solutions. Pharmacol Biochem Behav.

[38] Lardeux S, Kim JJ, Nicola SM (2013). Intermittent access to sweet high-fat liquid induces increased palatability and motivation to consume in a rat model of binge consumption. Physiol Behav.

[39] Robinson SL, McCool BA (2015). Microstructural analysis of rat ethanol and water drinking patterns using a modified operant self-administration model. Physiol Behav.

[40] Darevsky D, Gill TM, Vitale KR, Hu B, Wegner SA, Hopf FW (2019). Drinking despite adversity: behavioral evidence for a head down and push strategy of conflict-resistant alcohol drinking in rats. Addict Biol.

[41] Baird J-P, St. John SJ, Nguyen EA-N (2005). Temporal and qualitative dynamics of conditioned taste aversion processing: combined generalization testing and licking microstructure analysis. Behav Neurosci.

[42] Rhodes JS, Ford MM, Yu CH, Brown LL, Finn DA, Garland T (2007). Mouse inbred strain differences in ethanol drinking to intoxication. Genes Brain Behav.

[43] Wilcox MV, Carlson VCC, Sherazee N, Sprow GM, Bock R, Thiele TE (2014). Repeated binge-like ethanol drinking alters ethanol drinking patterns and depresses striatal gabaergic transmission. Neuropsychopharmacology.

[44] Salling MC, Skelly MJ, Avegno E, Regan S, Zeric T, Nichols E (2018). Alcohol consumption during adolescence in a mouse model of binge drinking alters the intrinsic excitability and function of the prefrontal cortex through a reduction in the hyperpolarization-activated cation current. J Neurosci.

[45] Linsenbardt DN, Boehm SL (2014). Alterations in the rate of binge ethanol consumption: implications for preclinical studies in mice. Addict Biol.

[46] Linsenbardt DN, Boehm SL (2015). Relative fluid novelty differentially alters the time course of limited-access ethanol and water intake in selectively bred high-alcohol-preferring mice. Alcohol Clin Exp Res.

[47] Ardinger CE, Grahame NJ, Lapish CC, Linsenbardt DN (2020). High alcohol-preferring mice show reaction to loss of ethanol reward following repeated binge drinking. Alcohol Clin Exp Res.

[48] Siciliano CA, Noamany H, Chang C-J, Brown AR, Chen X, Leible D (2019). A cortical-brainstem circuit predicts and governs compulsive alcohol drinking. Science.

[49] June HL, Gilpin NW (2010). Operant self-administration models for testing the neuropharmacological basis of ethanol consumption in rats. Curr Protoc Neurosci.

[50] Elmer GI, Meisch RA, George FR (1987). Differential concentration-response curves for oral ethanol self-administration in C57BL/6J and BALB/cJ mice. Alcohol.

[51] Elmer GI, Meisch RA, George FR (1987). Mouse strain differences in operant self-administration of ethanol. Behav Genet.

[52] George FR (1987). Genetic and environmental factors in ethanol self-administration. Pharmacol Biochem Behav.

[53] Risinger FO, Brown MM, Doan AM, Oakes RA (1998). Mouse strain differences in oral operant ethanol reinforcement under continuous access conditions. Alcohol Clin Exp Res.

[54] Grant KA, Samson HH (1985). Oral self administration of ethanol in free feeding rats. Alcohol.

[55] Grant KA, Samson HH (1986). The induction of oral ethanol self-administration by contingent ethanol delivery. Drug Alcohol Depend.

[56] Brown G, Jackson A, Stephens DN (1998). Effects of repeated withdrawal from chronic ethanol on oral self-administration of ethanol on a progressive ratio schedule. Behav Pharmacol.

[57] Jeanblanc J, Rolland B, Gierski F, Martinetti MP, Naassila M (2019). Animal models of binge drinking, current challenges to improve face validity. Neurosci Biobehav Rev.

[58] Green AS, Grahame NJ (2008). Ethanol drinking in rodents: is free-choice drinking related to the reinforcing effects of ethanol?. Alcohol.

[59] Juárez J, De Tomasi EB (1999). Sex differences in alcohol drinking patterns during forced and voluntary consumption in rats. Alcohol.

[60] Randall PA, Stewart RT, Besheer J (2017). Sex differences in alcohol self-administration and relapse-like behavior in long-evans rats. Pharmacol Biochem Behav.

[61] Vetter-O’Hagen CS, Varlinskaya E, Spear L (2009). Sex differences in ethanol intake and sensitivity to aversive effects during adolescence and adulthood. Alcohol Alcohol.

[62] Lancaster FE, Spiegel KS (1992). Sex differences in pattern of drinking. Alcohol.

[63] Lancaster FE, Brown TD, Coker KL, Elliott JA, Wren SB (1996). Sex differences in alcohol preference and drinking patterns emerge during the early postpubertal period in Sprague-Dawley rats. Alcohol Clin Exp Res.

[64] Gilpin NW, Karanikas CA, Richardson HN (2012). Adolescent binge drinking leads to changes in alcohol drinking, anxiety, and amygdalar corticotropin releasing factor cells in adulthood in male rats. PLoS ONE.

[65] Karanikas CA, Lu Y-L, Richardson HN (2013). Adolescent drinking targets corticotropin-releasing factor peptide-labeled cells in the central amygdala of male and female rats. Neuroscience.

[66] Hoffman JL, Faccidomo S, Saunders BL, Taylor SM, Kim M, Hodge CW (2021). Inhibition of AMPA receptors (AMPARs) containing transmembrane AMPAR regulatory protein γ-8 with jnj-55511118 shows preclinical efficacy in reducing chronic repetitive alcohol self-administration. Alcohol Clin Exp Res.

[67] Vendruscolo LF, Barbier E, Schlosburg JE, Misra KK, Whitfield TW, Logrip ML (2012). Corticosteroid-dependent plasticity mediates compulsive alcohol drinking in rats. J Neurosci.

[68] Wise RA (1973). Voluntary ethanol intake in rats following exposure to ethanol on various schedules. Psychopharmacologia.

[69] Simms JA, Steensland P, Medina B, Abernathy KE, Chandler LJ, Wise R (2008). Intermittent access to 20 % ethanol induces high ethanol consumption in long-evans and wistar rats. Alcohol Clin Exp Res.

[70] McHugh ML (2011). Multiple comparison analysis testing in anova. Biochem Medica.

[71] Armstrong RA (2014). When to use the bonferroni correction. Ophthalmic Physiol Opt.

[72] Wright PS. Adjusted p-values for simultaneous inference. Biometrics. 1992;1005–13. http://www-stat.wharton.upenn.edu/~steele/Courses/956/Resource/MultipleComparision/Writght92.pdf.

[73] Bekman NM, Winward JL, Lau LL, Wagner CC, Brown SA (2013). The impact of adolescent binge drinking and sustained abstinence on affective state. Alcohol Clin Exp Res.

[74] Kuntsche E, Müller S (2012). Why do young people start drinking? Motives for first-time alcohol consumption and links to risky drinking in early adolescence. Eur Addict Res.

[75] Orio L, Antón M, Rodríguez-Rojo IC, Correas Á, García-Bueno B, Corral M (2018). Young alcohol binge drinkers have elevated blood endotoxin, peripheral inflammation and low cortisol levels: neuropsychological correlations in women. Addict Biol.

[76] Moss-Racusin CA, Miller HG (2016). “Taking charge” of stigma: treatment seeking alleviates mental illness stigma targeting men. J Appl Soc Psychol.

[77] World Health Organization. Gender and women’s mental health. Ment Health Subst Use. https://www.who.int/teams/mental-health-and-substance-use/gender-andwomen-s-mental-health.

[78] Berman ME, Fanning JR, Guillot CR, Marsic A, Bullock J, Nadorff MR (2017). Effect of alcohol dose on deliberate self-harm in men and women. J Consult Clin Psychol.

[79] Townshend JM, Duka T (2005). Binge drinking, cognitive performance and mood in a population of young social drinkers. Alcohol Clin Exp Res.

[80] He J, Overstreet DH, Crews FT (2009). Abstinence from moderate alcohol self-administration alters progenitor cell proliferation and differentiation in multiple brain regions of male and female p rats. Alcohol Clin Exp Res.

[81] Cabral A, Isoardi N, Salum C, Macedo CE, Nobre MJ, Molina VA (2006). Fear state induced by ethanol withdrawal may be due to the sensitization of the neural substrates of aversion in the dpag. Exp Neurol.

[82] Avegno EM, Lobell TD, Itoga CA, Baynes BB, Whitaker AM, Weera MM (2018). Central amygdala circuits mediate hyperalgesia in alcohol-dependent rats. J Neurosci.

[83] Holleran KM, Winder DG (2017). Preclinical voluntary drinking models for alcohol abstinence-induced affective disturbances in mice. Genes Brain Behav.

[84] Morales M, McGinnis MM, McCool BA (2015). Chronic ethanol exposure increases voluntary home cage intake in adult male, but not female, long-evans rats. Pharmacol Biochem Behav.

[85] Almeida OFX, Shoaib M, Deicke J, Fischer D, Darwish MH, Patchev VK (1998). Gender differences in ethanol preference and ingestion in rats: the role of the gonadal steroid environment. J Clin Invest.

[86] Silva-Gotay A, Davis J, Tavares ER, Richardson HN (2021). Alcohol drinking during early adolescence activates microglial cells and increases frontolimbic interleukin-1 beta and toll-like receptor 4 gene expression, with heightened sensitivity in male rats compared to females. Neuropharmacology.

[87] Priddy BM, Carmack SA, Thomas LC, Vendruscolo JCM, Koob GF, Vendruscolo LF (2017). Sex, strain, and estrous cycle influences on alcohol drinking in rats. Pharmacol Biochem Behav.

[88] Bell RL, Rodd ZA, Sable HJK, Schultz JA, Hsu CC, Lumeng L (2006). Daily patterns of ethanol drinking in peri-adolescent and adult alcohol-preferring (p) rats. Pharmacol Biochem Behav.

[89] Blegen MB, da Silva E Silva D, Bock R, Morisot N, Ron D, Alvarez VA (2018). Alcohol operant self-administration: investigating how alcohol-seeking behaviors predict drinking in mice using two operant approaches. Alcohol.

[90] Richardson HN, Lee SY, O’Dell LE, Koob GF, Rivier CL (2008). Alcohol self-administration acutely stimulates the hypothalamic-pituitary-adrenal axis, but alcohol dependence leads to a dampened neuroendocrine state. Eur J Neurosci.

[91] Gilpin NW, Smith AD, Cole M, Weiss F, Koob GF, Richardson HN (2009). Operant behavior and alcohol levels in blood and brain of alcohol-dependent rats. Alcohol Clin Exp Res.

[92] Teschke R, Wiese B (1982). Sex-dependency of hepatic alcohol metabolizing enzymes. J Endocrinol Invest.

[93] Quintanilla ME, Tampier L, Sapag A, Gerdtzen Z, Israel Y (2007). Sex differences, alcohol dehydrogenase, acetaldehyde burst, and aversion to ethanol in the rat: a systems perspective. Am J Physiol Metab.

[94] Shukla SD, Restrepo R, Aroor AR, Liu X, Lim RW, Franke JD (2019). Binge alcohol is more injurious to liver in female than in male rats: histopathological, pharmacologic, and epigenetic profiles. J Pharmacol Exp Ther.

[95] Marsland P, Parrella A, Vore AS, Barney TM, Varlinskaya EI, Deak T (2021). Male, but not female, Sprague Dawley rats display enhanced fear learning following acute ethanol withdrawal (hangover). Pharmacol Biochem Behav.

[96] Li TK, Beard JD, Orr WE, Kwo PY, Ramchandani VA, Thomasson HR (2000). Variation in ethanol pharmacokinetics and perceived gender and ethnic differences in alcohol elimination. Alcohol Clin Exp Res.

[97] Kwo PY, Ramchandani VA, O’Connor S, Amann D, Carr LG, Sandrasegaran K (1998). Gender differences in alcohol metabolism: relationship to liver volume and effect of adjusting for body mass. Gastroenterology.

[98] Grittner U, Wilsnack S, Kuntsche S, Greenfield TK, Wilsnack R, Kristjanson A (2020). A multilevel analysis of regional and gender differences in the drinking behavior of 23 countries. Subst Use Misuse.

[99] Dettling A, Fischer F, Böhler S, Ulrichs F, Skopp G, Graw M (2007). Ethanol elimination rates in men and women in consideration of the calculated liver weight. Alcohol.

[100] Dettling A, Skopp G, Graw M, Haffner HT (2008). The influence of sex hormones on the elimination kinetics of ethanol. Forensic Sci Int.

[101] Cole-Harding S, Wilson JR (1987). Ethanol metabolism in men and women. J Stud Alcohol.

[102] Müller C (2006). Liver, alcohol and gender. Wien Med Wochenschr.

[103] Lieber CS (2000). Ethnic and gender differences in ethanol metabolism. Alcohol Clin Exp Res.

[104] Vargas WM, Bengston L, Gilpin NW, Whitcomb BW, Richardson HN (2014). Alcohol binge drinking during adolescence or dependence during adulthood reduces prefrontal myelin in male rats. J Neurosci.

[105] Sneddon EA, Ramsey OR, Thomas A, Radke AK (2020). Increased responding for alcohol and resistance to aversion in female mice. Alcohol Clin Exp Res.

[106] Szumlinski KK, Coelho MA, Lee KM, Tran T, Sern KR, Bernal A (2019). DID it or didn’t it? exploration of a failure to replicate binge-like alcohol-drinking in c57bl/6j mice. Pharmacol Biochem Behav.

[107] Rhodes JS, Best K, Belknap JK, Finn DA, Crabbe JC (2005). Evaluation of a simple model of ethanol drinking to intoxication in c57bl/6j mice. Physiol Behav.

[108] Thiele TE, Crabbe JC, Boehm SL (2014). “Drinking in the dark” (DID): a simple mouse model of binge-like alcohol intake. Curr Protoc Neurosci.

[109] Lei K, Wegner SA, Yu J-H, Hopf FW (2016). Orexin-1 receptor blockade suppresses compulsive-like alcohol drinking in mice. Neuropharmacology.

[110] Thiele TE, Navarro M (2014). “Drinking in the dark” (DID) procedures: a model of binge-like ethanol drinking in non-dependent mice. Alcohol.

[111] Yoshida M, Koyanagi S, Matsuo A, Fujioka T, To H, Higuchi S (2005). Glucocorticoid hormone regulates the circadian coordination of μ-opioid receptor expression in mouse brainstem. J Pharmacol Exp Ther.

[112] Atkinson HC, Waddell BJ (1997). Circadian variation in basal plasma corticosterone and adrenocorticotropin in the rat: sexual dimorphism and changes across the estrous cycle*. Endocrinology.

[113] Anthenelli RM, Heffner JL, Blom TJ, Daniel BE, McKenna BS, Wand GS (2018). Sex differences in the acth and cortisol response to pharmacological probes are stressor-specific and occur regardless of alcohol dependence history. Psychoneuroendocrinology.

[114] Lu Y-L, Richardson HN (2014). Alcohol, stress hormones, and the prefrontal cortex: a proposed pathway to the dark side of addiction. Neuroscience.

[115] Rivier C (1993). Female rats release more corticosterone than males in response to alcohol: influence of circulating sex steroids and possible consequences for blood alcohol levels. Alcohol Clin Exp Res.

[116] Carnicella S, Amamoto R, Ron D (2009). Excessive alcohol consumption is blocked by glial cell line-derived neurotrophic factor. Alcohol.

[117] Crabbe JC, Metten P, Rhodes JS, Yu C-H, Brown LL, Phillips TJ (2009). A line of mice selected for high blood ethanol concentrations shows drinking in the dark to intoxication. Biol Psychiatry.

[118] Baliño P, Romero-Cano R, Sánchez-Andrés JV, Valls V, Aragón CG, Muriach M (2019). Effects of acute ethanol administration on brain oxidative status: the role of acetaldehyde. Alcohol Clin Exp Res.

[119] Israel Y, Quintanilla ME, Karahanian E, Rivera-Meza M, Herrera-Marschitz M (2015). The “first hit” toward alcohol reinforcement: role of ethanol metabolites. Alcohol Clin Exp Res.

